# Covid-19 pandemic: our relationships, environment, and health

**DOI:** 10.14324/111.444/ucloe.000045

**Published:** 2022-09-16

**Authors:** Keri Ka-Yee Wong

**Affiliations:** 1 Department of Psychology and Human Development, University College London, London, UK

Human health is intimately linked with the environment [1]. This includes the spaces in which one lives, learns and works. All of the key environments in which we occupy and interact with drastically changed when the World Health Organisation (WHO) [2] declared Covid-19 a pandemic in March 2020 and when subsequent government lockdowns and other physical restrictions were imposed. Our relationship with our environment changed suddenly and in unexpected ways – and may never be quite the same again.

In broad terms, these changes to the nature of the environment in which people live, learn and work came about as a consequence of pandemic control measures. To minimise the impact of a new, very infectious disease people were asked or required to remain at home, to work from home (if possible), to maintain a distance between themselves and others and to limit use of outside spaces, say, for exercise use only. In many countries, schools and universities were closed, education moved online and this meant many families were faced with real issues about how to balance online work with the need to ensure online education happened as well. Travel was restricted nationally and internationally, and a range of legal measures were introduced to enforce compliance. In many countries, people were asked to wear masks indoors and in public places for the first time which introduced a new social norm.

In this series on Covid-19 and Mental Health published in the journal *UCL Open: Environment* (see https://doi.org/10.14293/S2199-1006.1.SOR-EARTH.CLIEN0H.v1), we highlight findings from the UCL–Penn Global Covid Study, a 12-month longitudinal study of the impact of Covid-19 on social trust, mental health and physical health, launched in April 2020 [3]. In collaboration with six institutions from Italy, Singapore, the USA, China and the UK, the study looks at the short- and longer-term effects of Covid-19 on individual’s mental health and social relationships with others by collecting time-sensitive survey data to understand how a large-scale change in the global environment can have impacts on health during a pandemic’s early stages.

The series, funded by the UCL Global Engagement Fund, examines some of the linkages between people’s physical and social environment and their mental health that have been revealed during the Covid-19 pandemic. Some of these findings will likely read across to the journal’s broader special series on Covid-19 interactions with our environment (https://ucl-about.scienceopen.com/covid19-specialseries) to facilitate interdisciplinary discussions concerned with the persistent global impact of the Covid-19 pandemic.

In addition, this series also publishes alongside the research articles, ‘discussant’ articles recording the policy relevance and study implications about the lessons learned of the impact on our mental health of the Covid-19 driven change in our environment. Provided by invited policy makers and other subject experts attending the Study’s online summer webinar series which was held online between the 2nd June and the 28th July, 2021 and composed of five online webinars (one webinar covering each of the series articles). This is a first and novel attempt at this new journal format to publish research project findings alongside recorded implications and the possible impact of it.

The Covid-19 pandemic is a naturalistic stressor that has disrupted our home, leisure, work and educational lives. This deadly airborne ‘invisible’ virus that survives in the spaces we live, learn and occupy has caused psychological distress and uncertainty to individuals because the virus does not discriminate based on ethnicities, background or age. The lead question for this series was: how has the Covid-19-altered environment impacted health and relationships?

In each of the five articles in this series, we discuss the impact and consequences that environmental changes brought about by the Covid-19 pandemic has triggered forced government lockdown restrictions and the impacts that these have had on our health and relationships.

The study of coronavirus and the associated environmental consequences of government responses in terms of lockdowns and restrictions is of vital importance in thinking about where resources should be deployed for pandemic recovery. What is clear in this pandemic is how Covid-19 has changed the various environments in which we occupy, play, live, learn and no longer have access to – and the consequences this has on our health and relationships. Particularly, the health inequalities, mental health access and support issues, technology gap between social strata, cuts in funding for third-sector support groups and structural inefficiencies in various sectors – that have traditionally been brushed under the rug before the pandemic – have been brought into sharp focus during this pandemic.

A central and integral question to humans as a social species is what impact does the lack of social contact as a consequence of forced Covid-19 lockdown restrictions have on our physical and mental health? Unsurprisingly, loneliness emerges as an important factor in more than one way as reported in the series articles. For example, in Carollo et al. [4], the authors used novel machine learning techniques to understand the impact of lockdown duration and restrictions on adults from Greece and the UK and revealed that loneliness and depression were key symptoms that fluctuated during the pandemic. Even prior to the pandemic, there were discussions about a worldwide ‘loneliness epidemic’. Kasley Killam, founder of Social Health Labs, in a discussant article [5], recognised the need to facilitate initiatives to tackle loneliness as part of ‘social’ health and offered insights into existing local, national and international initiatives in place to reconnect individuals. Social health, she argues, is of equal importance to mental and physical health, and perhaps a priority area for post-pandemic recovery strategies when considering the importance of local community spaces in promoting social connections in the population and pandemic recovery.

The wealth of initiatives tackling global loneliness to increase our social health is further elaborated on in Wong et al. [6]. Here, the authors found that individuals’ feelings of loneliness were centrally linked to distrust in others in the community and also depressive symptoms, which in turn were associated with other mental health symptoms (e.g., anxiety, stress about covid, insomnia). Trust in the community has always been pivotal to facilitating community peace and cohesion in society. Interestingly, but perhaps not surprisingly, a discussant article by Mitch Cooke (Head of Sustainability, Greengage Environmental) observed a shift in focus on people’s attitudes towards modifying the immediate home environment – as people are now spending more time ‘working from home’ – and ensuring these are now compatible multifunctioning workspaces [7]. He discusses how this shift in people’s needs can impact architectural decisions to an emphasis on ‘greening’ the indoor home space (previously more of a focus on outdoor green spaces) as potential environmental decisions to improving health. Conversely, Dr Emma Barkus, (Associate Professor, University of Northumbria) discusses the short and longer-term advantages and disadvantages of loneliness [8]. This is a state that is evolutionarily advantageous as it reminds us to reconnect with our community, socially, but failure to do so, perhaps in changing our environments, may mean that feelings of self-perceived loneliness can persist and in turn impact our personality and the way in which one views the world. It is clear that the Covid-19 pandemic restrictions have blurred the line between indoor ‘home’ space as one for relaxation and a space to retreat, with other spaces associated with work, play and education. Many individuals and families have had to carefully negotiate, and in some cases redesign, their relationship with their home environments.

Our series of studies also examined subpopulations and the micro-environments in which they operate in. How has the Covid-19 restrictions, which dictated who and which environments we can or cannot access, impacted vulnerable groups who may be living in more complicated home environments? In Portnoy et al. [9], we investigate whether the Covid-19 ‘stay at home’ order was particularly challenging for families with young children, and how this relates to parent’s mental health using transactional models applied on two waves of strict lockdown periods. By accounting for both parent-effects and child-effects across timepoints, the authors begin to understand how the pandemic household environment can change family dynamics over time. The idea that the important relational interactions between family members may be impacted within the confines of the home when families are spending everyday of every week under the same roof. This is contextualised within possible policy implications in government support for families with young children as discussed by Dr Yahayra Michel (School of Criminology and Justice Studies, University of Massachusetts Lowell, USA) [10]. Dr Michel highlights the complexity of family systems and potential family interventions ought to be multifaceted and working across disciplines and agencies and available to both children and parents during times of challenge. For us to be better prepared for future pandemics and should work from home mandates become more commonplace, better communicative and collaborative working relationships need to be in place now (e.g., with social services, families, children and schools).

A similar conversation was had when thinking about students. In spaces of learning, such as universities, access to resources and mentors had become limited and research student projects were often grounded to a halt, if not moved to a virtual environment. In Sideropoulos et al. [11], the authors examine the wellbeing of doctoral students (i.e., PhD students) to examine how the cumulative stressors resulted from the Covid-19 pandemic may explain student’s health, especially when a high proportion live with existing mental health issues already. For the doctoral student community, the cumulative stressful events experienced at the macro–meso–micro levels and the perceived uncertainties during the Covid-19 pandemic increased levels of depressive symptoms (not anxiety), which was particularly true of individuals with poorer coping and attentional skills – potential areas of intervention. These findings were not unique to students in higher education but were also replicated in international studies of non-WEIRD (Western, Educated, Industrialized, Rich and Democratic) societies as Tara Beteille (Senior Economist, The World Bank) points out in her discussant article on schoolteachers, children and support [12]. The Covid-19 pandemic forced educators and students to negotiate the balance between online and offline learning environments and our patience as well as motivation to stay ‘connected’ albeit virtually. This area of research extends the longstanding debates about the efficacy of hybrid models of learning (e.g., distance learning) and, post-pandemic, challenges the strengths and limitations of virtual vs face-to-face learning environments.

It is no surprise that lockdown restrictions – a legally imposed physical detachment from others aimed to keep everyone safe in order to decrease coronavirus transmission in the community – have taken a toll on everyone. In Wong et al. [13], the authors present a rich qualitative summary of the variety of impacts that study respondents have experienced when reflecting on the pandemic 12 months after. Crucially, the authors also identified the variety of support that respondents would need in order to recover. These included beyond financial support, but continued mental health access, flexible working and childcare support, and technology training and access. These comments were echoed by Deborah Alina (MBE and CEO, Independent Age) and Morgan Vine’s findings on the mental health of elderly populations, who are most often neglected in public discourse and living in environments that were not covid-safe [14]. Through their research, they found that lockdown restrictions meant that elderly populations faced extremely challenging situations during the pandemic as their lack of access to technology combined with poor physical connection also took a toll on people’s wellbeing. Professor David Murphy (Former President and Covid Response Lead, British Psychological Society) [15] and Nigel Atter (Policy Advisor, British Psychological Society) [16] shared their perspectives from the British Psychological Society – the society for psychologists and psychology in the UK. Both speakers spoke about the numerous supports offered during the pandemic in the form of psychological helplines, knowledge for the public (e.g., coping strategies, psychological workshops and online resources), as well as new policy recommendations which were taken by the society to better inform the government and organisations on their Covid response and recovery strategy. It remains to be seen whether major changes to the environment can indeed facilitate better health and recovery in the months to come. The articles in this series do highlight a few key areas of focus for recovery plans.

Evidently, this series captures the ongoing changing environment due to the Covid-19 pandemic and associated government restrictions on individual’s and group’s health (physical, mental and social). This huge change to the environments in which we live, play, work and learn, has had notable changes in our health, and we are far from completely understanding what the short- and longer-term effects of the pandemic will be. However, a consistent theme throughout all publications in academia and industry, is the increasing need to come together as disciplines, to converse, to share good practices and co-create workable solutions for all in a mindful way. There is existing support for mental health at both the community and national level. Instead of cutting funding for local third sectors who are doing good work, we need to resource those and ensure they are well supported now and in time for future pandemics. Educating the population through clear messaging around expectations during the pandemic in terms of mental health can help individuals and families cope with and better manage symptoms fluctuations. Reasonable policies around time spent outdoors, even if brief, may have considerable impacts on individual’s health and wellbeing and more widely. But most importantly of all, this series has highlighted the importance of bringing different perspectives to the same table, as we design strategies to recover, be more resilient, and build back better.
